# Post subthalamic area deep brain stimulation for tremors: a mini-review

**DOI:** 10.1186/2047-9158-1-20

**Published:** 2012-10-29

**Authors:** Tao Xie, Jacqueline Bernard, Peter Warnke

**Affiliations:** 1 Department of Neurology, University of Chicago Medical Center, Chicago, IL, 60637, USA; 2 Department of Neurosurgery, University of Chicago Medical Center, Chicago, IL, 60637, USA

**Keywords:** Post subthalamic area, Zona incerta, Deep brain stimulation, Tremor

## Abstract

Deep brain stimulation (DBS) in the thalamic ventrointermediate nucleus (VIM) is the traditional target for the surgical treatment of pharmacologically refractory essential tremor or parkinsonian tremor. Studies in recent years on DBS in posterior subthalamic area (PSA), including the zona incerta and the prelemniscal radiation, have shown promising results in tremor suppression, particularly for those tremors difficult to be well controlled by VIM DBS, such as the proximal postural tremor, distal intention tremor and some cerebellar outflow tremor in various diseases including essential tremor and multiple sclerosis. The adverse effect profile of the PSA DBS is mild and transient, without lasting or striking dysarthria, disequilibrium or tolerance, in contrast to VIM DBS, particularly bilateral DBS. However, the studies on PSA DBS so far are still limited, with a handful of studies on bilateral PSA, and a short follow up duration compared to VIM. More studies are needed for direct comparison of these targets in the future. A review here would help to gain more insight into the benefits and limits of the PSA DBS compared to that in VIM in the clinical management of various tremors, particularly for those difficult to be well controlled by traditional VIM DBS.

## Introduction

Deep brain stimulation (DBS) in thalamic ventrointermediate (VIM) nucleus is the traditional target for the surgical treatment of pharmacologically refractory essential tremor (ET) or parkinsonian tremor. Studies in recent years on DBS in the posterior subthalamic area (PSA), including the zona incerta (Zi) and the prelemniscal radiation (Raprl), have shown promising results in tremor suppression [1-25], particularly for those difficult to be controlled by VIM DBS, such as the proximal postural tremor, distal intention tremor and some cerebellar outflow tremor in ET, multiple sclerosis (MS), post-traumatic tremor (PTT), cerebellar tremor (CT), Holmes tremor (HT) and spinocerebellar ataxia 2 (SCA2) [8,12-14,16]. The adverse effect profile of the PSA DBS is mild and transient, without lasting or striking dysarthria, disequilibrium or tolerance, as would have been seen in the VIM DBS, particularly bilateral DBS [26-30]. However, the studies on PSA are still limited given less than 30 publications in the PubMed so far, with even a handful of studies performed on bilateral PSA, and a short follow up duration compared to VIM. Therefore, a mini-review on DBS in PSA is needed to gain more comprehensive insight into the potential benefits and limits of the PSA DBS compared to that in VIM DBS in the clinical management of various tremors, particularly for those tremors difficult to be well controlled by traditional VIM DBS.

## DBS in PSA: evidence on effective tremor control and others

Anatomically, the PSA is bounded anteriorly by the posterior border of the subthalamic nucleus (STN), superiorly by the ventral thalamic nuclei, inferiorly by the dorsal border of the substantia nigra, posteriorly by the medial lemniscus, posteromedially by the anterolateral border of the red nucleus, posterolaterally by the ventrocaudal nucleus, and laterally by the posterior limb of the internal capsule [31]. It consists of Zi and the Raprl. The Zi lies dorsal and posterior to STN, joining both the basal gangalia thalamocortical circuit and the cerebellar thalamocortical circuit. The Zi anatomically also consists of caudal part and rostral part. The Raprl is a fiber bundle that lies posterior to the STN, separated from it by the intervening Zi. It contains fibers from the mesencephalic reticular formation that projects to the thalamus as well as ascending cerebellothalamic fibers. The Forel H1 (thalamic fasciculus) and H2 (lenticular fasciculus) lies dorsal to the STN and immediately anterior to the PSA, and the Forel H lies anterior to the red nucleus.

Whilst the Zi was a target for lesioning since the early sixties, DBS in PSA was first reported by Mundinger in 1977 [1]. The intraoperative stimulation of the Zi and surrounding area obtained good control of torticollis in 7 patients without complications [1]. Successful results were also reported by Brice and McLellan in 1980 and Andy in 1983 [2,3]. The unsophisticated DBS devices, the successful therapeutic effects of levodopa in Parkinson’s disease (PD), and the successful DBS on VIM by Benabid in 1987 [32] are all possible reasons for the lack of sufficient reports during that period of time. In 2000, a successful attempt on the DBS targeting Zi in controlling intention tremor in ET and dystonic tremor (DT), which were hard to be controlled otherwise by DBS targeting VIM, was reported by Kitagawa et al. [4]. This study reignited the interest of DBS targeting Zi and more broadly PSA. Since then, more than 20 articles have been published, as listed in the Table 1, including 201 cases of ET, 99 cases of PD, 32 cases of MS, 6 cases of DT, and several others (PTT, CT, HT, WC, and SCA) [1-25]. The majority of the DBS was placed unilaterally. Only about 25% of the cases had bilateral DBS (Table 1). They were followed up for 3 months to 6 years. Most of them were targeting caudal Zi (cZi), with different parameters among studies. The therapeutic benefit was prominent, particularly for those tremors difficult to be controlled by VIM, such as proximal postural tremor, distal intension tremor and cerebellar out flow tremor. The complications mostly were mild and transient. There was no lasting or striking dysphasia, dysarthria, or disequilibrium, which could be encountered in VIM DBS, particularly bilateral VIM DBS [26-29]. There was no tolerance either [9,25], another unfavorable feature often reported in VIM DBS [30]. The effect on axial symptoms, such as vocal tremor, head tremor and swallowing function, was rarely mentioned [9]. A good effect on neck tremor was reported on bilateral cZi DBS [9]. DBS on cZi was not found to have negative effect on the swallowing function [33], but have slightly different effect on voice compared to STN DBS [34].

**Table 1:** PSA DBS publications: indications, targets, results and side effects

**Series (reference number)**	**Patients/procedures/time to assess**	**Target and/or stereotactic parameters**	**Results**	**Side effects**
Mundinger, 1977 [1]	7 torticollis, unilateral, stimulation 30-40 minutes.	cZi; in some cases combined with other structures	Good control of the torticollis	No
Brice and McLellan, 1980 [2]	2 MS, bilateral, post-op 6 months	10mm lateral/20mm behind AC/6–8mm below ICL (AC: anterior commissure; ICL: inter-commissural line)	“Striking improvement” in intention tremor	Transient worsening of swallowing, speech, and micturition, all resolved in 3 weeks but dysarthria.
Andy, 1983 [3]	1 PTT, unilateral	7mm lateral/ 8.5mm behind MCP/1mm below ICL (MCP: middle-commissural point)	Complete cessation of tremor	Unknown
Kitagawa et al., 2000 [4]	1 ET and 1 DT, unilateral, intra-op stimulation and post-op 1 week	Zi, 3 mm under the border of the VIM	Abolition of ET; “remarkable” decrease in DT and dystonia	Transient paresthesia, palm hyperhidrosis, anorexia, and disequilibrium
Hooper et al., 2001 [5]	1 PTT, unilateral, post-op 44 months	12mm lateral/ 6mm behind MCP/4mm below ICL	Sustained microtomy effect. No IPG needed.	Shoulder weakness, resolved in 3 days.
Velasco et al., 2001 [6]	10 PD, unilateral, post-op 12 months	Expressed in tenths of the ICL: laterality 5/10, 8/10 behind AC, 1–2/10 below ICL, targeting Raprl	Significant improvement in tremor and rigidity; Mild improvement in bradykinesia.	1 worsening pre-existing depression, 1 transient diplopia, 3 transient dysarthria
Murata et al., 2003 [7]	8 ET, unilateral, post-op 22 months (8-42)	Best 11mm lateral/7.5mm behind MCP/4mm below ICL in Zi and Raprl	Contralateral tremor decreased by 81%	Only stimulation induced that did not affect result.
Nandi and Aziz, 2004 [8]	15 MS, 6 bilateral, 9 unilateral, post-op 15 months in 10 patients	Zi	Contralateral postural tremor decreased by 64%, intention tremor by 36%	Transient paresthesia, mild dysarthria and seizure in 1 and infection in 2 patients.
Plaha et al., 2004 [9]	4 ET, bilateral, post-op 12 months	Medial to the posterior dorsal third of the STN	Total tremor decreased by 80%. 2 patients with severe head tremor completely resolved. No tolerance. Low volt 1.8.	No dysarthria or dysequilibrium.
Kitagawa et al., 2005 [10]	8 PD, unilateral, post-op 24 months	Best contact 10.5mm lateral/5.6mm behind MCP/ 3.2mm below ICL	UPDRS-III improved by 44.3%, tremor by 78.3%, rigidity by 92.7% and akinesia by 65.7%.	Mild adverse events
Plaha et al., 2006 [11]	35 PD, 29 bilateral, 6 unilateral, post-op 6 months	cZi: posteromedial to the post-dorsal STN	cZi better than STN in reducing UPDRSIII by 76%, tremor by 93%, rigidity by 76% and bradykinesia by 65% in cZi vs by 55%, 61%, 50% and 59% in STN.	No complication in Zi No difference in dyskinesia, L-dopa reduction, and stimulation parameters.
Freund et al., 2007 [12]	1 SCA2, bilateral, post-op 2 years	Combined VOP-VIM/Zi-Cerebellar thalamic projection (VOP: ventro-oralis posterior).	Nearly complete cessation of tremor and torticollis by stimulation to distal contacts	No complication mentioned
Hamel et al., 2007 [13]	8 ET, 2 MS, 1 SCA, bilateral, post-op at least 3 months, most of them > 1year	12.7mm lateral/7mm behind MCP/1.5mm below ICL	Reducing intention tremor by 68% to 73%. PSA better than VIM unless limited by side effects	Paresthesia, dysarthria, gait ataxia, unknown number
Herzog et al., 2007 [14]	10ET, bilateral, and 11MS, 6 bilateral, 5 unilateral, post-op at least 4 months	In PSA region, no details	PSA better than VIM in postural and intention tremors reduction, by 64% in ET and by 50% in MS.	Unknown
Carrillo-Ruiz et al., 2008 [15]	5 PD, bilateral, post-op 12 months	Active contacts: 11.5mm/ 6.5mm behind MCP and 4.5mm below ICL	UPDRS III decreased by 65%, tremor by 90%, rigidity by 94%, bradykinesia by 75%	1 deterioration of pre-existing depression, 5 transient somnolence, 1 transient dysarthria
Plaha et al., 2008 [16]	6 ET, 5 PD, 4 MS, 1 CT, 1 HT, 1 DT/bilateral, post-op 12 months	Posteromedial to the posterodorsal STN	PD tremor improved by 92%, rigidity by 77%, bradykinesia by 62%. Tremor improved in ET by 76%; MS, 57%; CT, 60%; HT, 70%; DT, 71%. Low volts	2 transient dysequilibrium, 1 transient dysphagia
Blomstedt et al., 2009 [17]	2DT,1 WC (writer's cramp),1CT, all unilateral, post-op 1 year	Active 10.3mm/6.1mm behind MCP/3.5 below ICL, in PSA	87% tremor reduction	Unknown
Blomstedt et al., 2010 [18]	21ET, 2 bilateral, 19 unilateral, post-op 1 year.	PSA active contact 11.6mm lateral/6.3mm behind MCP/3mm below ICL.	Reducing tremor of upper extremity by 95%, hand function by 87%, improving ADL by 66%.	8 transient expressive dysphasia, 1 transient clumsy hand and leg.
Fytagoridis and Blomstedt, 2010 [19]	27 ET, 8 PD, 2 DT, 1 CT, 1 WC, all unilateral except 4 bilateral, unknown disease, post-op 34 months	Active 12.0mm/6.1mm behind MCP/1.5mm below ICL, all in PSA	24 non-PD tremor decreased by 91%	1 transient hemiparesis, 1 infection, 22% transient dysphasia.
Barbe et al., 2011 [20]	21ET, bilateral 19, 2 unilateral, post-op at least 3 months	26 sub- ICL and 14 above ICL electrodes. The mean sub-ICL 11.3mm lateral/7.2mm behind MCP/1.4mm below ICL, the thalamic 12.6mm lateral/5.7mm behind MCP/1.0mm above ICL.	Sub-ICL stimulation is more efficient than thalamic stimulation but equally effective when patients’ individual stimulation parameters are used.	Paresthesia in 3/26, and dysarthria in 2/26 electrodes
Blomstedt et al., 2011 [21]	4 ET unilateral, one in STN one in cZi, post-op 1-6 years	cZi 9.5-15.5mm lateral/1.3-9.4mm behind MCP/0.2mm above to 6.8mm below ICL	cZi more efficient than STN	Comparable, dysarthria, dystonia, dizziness, blurred vision.
Blomstedt et al., 2011 [22]	5ET, failed VIM, no info on post-op duration except in “years”	cZi, 11.4mm lateral/6.8mm behind MCP/2.9mm below ICL	cZi achieved improvement in tremor control after VIM failed, 57% cZi vs 25% VIM	Unknown
Blomstedt et al., 2011 [23]	68 ET, 34VIM and 34 PSA, only 3 each bilateral, post-op 28 months for VIM and 12 month for PSA.	Vim 13-15mm lateral/6-7mm before PC/0mm on ICL. PSA: posteromedial to the tail of the STN at the level of maxim diameter red nucleus (PC: posterior commissure)	Tremor in the treated hand improved by 70% in VIM and 89% in PSA.	Unknown
Blomstedt et al., 2012 [24]	14 PD, 13 unilateral, 1 bilateral, post-op 18 months	Posterior and medial to the posterior tail of the STN at the maximal diameter of the RN. Active contact 12.6mm lateral/7mm post MCP/2mm below ICL	Tremor reduction by 82.2%, rigidity by 34.3%, bradykinesia by 26.7%	1 stimulation induced side effect, 1 infection
Fytagoridis et al., 2012 [25]	18 ET, 16 unilateral and 2 bilateral, post-op 4 years on average	cZi, 12.0mm lateral/6.3mm behind MCP/2.2mm below ICL, in posterior-medial to STN at the level of the maximal diameter of red nucleus	Improved total tremor by 51.4%, upper extremity by 89.4%, hand function by 78.5%. No increase in stimulation over the course	Mild and transient, 1 hard ware related.

The difference in the specific targets of PSA, the number of DBS placed (unilaterally vs bilaterally), the post-surgical follow -up, and the different diseases studied among these articles makes a concise comparison of clinical benefits and limits of PSA with VIM difficult. A site-to-site comparison among different patients could be more helpful to delineate the difference between the targets. One of the good examples was the study by Hamel et al. [13], who found that cZi DBS at the parameters of 12.7 mm lateral, 7.0 mm posterior and 1.5 mm ventral to the mid-commisural point (MCP) provided better outcome than VIM DBS. Though it is a retrospective study with different post-surgical duration, the site-to-site comparison did provide more reliable comparison of these two targets. A similar result was also reported by another group [11,20,21], with electrodes below the intercommissural line (ICL) producing better outcome in tremor control than those above the ICL [20], though the better outcome meant to be more efficient for cZi than VIM rather than significant difference in individual or maximal stimulation response in these two targets in some studies [20]. More recently, a prospective study designed for direct comparison of PSA/cZi with VIM in each individual patient was presented to the 16^th^ MDS meeting in Dublin [35]. The electrode in the study was placed across both PSA/cZi and VIM, which would allow precisely comparing these two targets in the same patient, with both targets targeted in the same side of the brain, or with one target in one side of the brain and the other target in the other side of the brain of the same patient. This design avoids the confounding difference in the disease severity, post-surgical duration, and skills/targets in assessing the DBS efficacy, and allows more accurate site specific assessment (Zi vs VIM). A significantly better outcome in general was found in stimulating cZi than VIM, except that some of the patients could not tolerate the adverse effects of paresthesia in cZi despite the best control of the tremor. The vocal tremor and head tremor were also very well controlled by the stimulation. No worsening of gait was observed after the surgery. The study shows very promising tremor control by cZi DBS, though the conclusion is limited by the small sample size of five patients, as acknowledged by the authors [35]. Stimulating bilateral VIM was also reported to improve vocal tremor and head tremor in some studies as reviewed by Lyons and Pahwa [36], however the occurrence and the worsening of dysarthria and disequilibrium were the concerns in choosing bilateral VIM stimulation in some cases [26-29], and made unilateral VIM DBS as an alternative in certain circumstances [37]. The lack of lasting dysarthria and disequilibrium reported even in bilateral cZI DBS is probably because the cZi DBS only overrides tremor oscillations without interrupting patterns of information related to fine movements of vocal cords and proprioceptive sensation [16].

Besides effective tremor control [11,21,24], cZi was also found to be better than STN in controlling rigidity and bradykinesia without difference in reducing dyskinesia and levodopa equivalent dose [11]. The rigidity and bradykinesia was not found to be very well controlled by cZi DBS in another study though [24]. Dystonia was also found to be well controlled by cZi DBS [1,16,17]. More recently, cZi in combination with pedunculopontine nucleus was found to have positive effect on axial symptoms [38]. cZi DBS could also be a target for some patients with ataxia [39].

## DBS in PSA: targeting the targets

As neither cZi nor Raprl can be reliably distinguished on 1.5 Tesla MRI, some used the neighboring structures as reference, such as the STN or VIM, to guide the DBS placement. Most of the studies used posterior and medial to the posterior tail of the STN at the maximal diameter of the red nucleus as the target (Table 1). Some of them used the 2-3mm under the ventral border of the VIM as the target [4,35]. Only a few studies also used microelectrode recording to guide the electrode placement, as cZi gives silent or low activity neuronal background [35], which differentiates cZi from VIM. Imaging targeting in combination with macrostimulation was applied virtually by all studies.

Specifically, the PSA was identified on trans-axial T2-weighted MRI images slightly posterior medial to the subthalamic nucleus at the level of the maximal diameter of the red nucleus (Table 1), or about 2-3mm below the ventral border of the VIM [4,35]. A pre-operative MRI fused with a head CT with stereotactic frame and superimposed digitized Schaltenbrand stereotactic atlas was used to plan the trajectory. The electrode was implanted under local anesthesia. EMR was further used in some studies [35]. The final position of the electrode was dictated by the response to the macrostimulation. A postoperative x-ray was performed before removal of the frame. The location of each electrode contact post-surgically was determined on the postoperative CT infused with the pre-surgical MRI and superimposed atlas. The DBS contact location was determined in relation to the anterior commissure (AC) – posterior commissure (PC) line and the coordinates were plotted onto the Schaltenbrand stereotactic atlas. The efficacy of the stimulation was presented as the result on stimulation in relation to the result off stimulation at the same evaluation, based on clinical exam, UPDRS motor scores, tremor rating scores, daily living function, or quality of life assessment.

## DBS in PSA: possible mechanism

The mechanism of tremor suppression by stimulation in PSA (mostly in cZi) is not entirely clear. The Zi is a heterogenous nucleus that lies at the base of the dorsal thalamus and is an extension of the reticular /thalamic nucleus [40]. Its rostral component extends over the dorsal and medial surface of the STN, and its caudal or motor component lies posteromedial to the STN [41,42]. The ZI receives afferents from the globus pallidus internus (GPi), the substantia nigra reticulate (SNr) [41,43,44], the ascending reticular activating system [43-45], the interpositus nucleus of the cerebellum, and also the motor, associative and limbic areas of the cerebral cortex [43,46]. The ZI sends efferents to the centromedian and parafascicular nuclei [47-49], the ventral anterior nucleus and the ventral lateral nucleus of the thalamus [50], the midbrain extrapyramidal area and the medial reticular formation [41], the GPi and SNr [41], the interpositus nucleus of the cerebellum, the inferior olive and cerebral cortex [51-53]. Current hypotheses regarding the mechanisms of tremor generation point to abnormal synchronisation of neuronal firing in the basal ganglia thalamocortical loop (in PD and DT) or the cerebellar thalamocortical loop (in ET, CT and MS tremor) or both loops (HT) [16]. cZi is an effective target for the surgical control of all forms of tremor because of its unique GABAergic connections with both the basal ganglia and cerebellar thalamocortical loops, in addition to the brain stem motor effectors through which tremor oscillation may be transmitted [16]. Stimulation of the Zi is likely to suppress the tremor by overriding the oscillations in these areas [16]. Stimulation of the Raprl could similarly abolish contralateral tremor and reduce rigidity [54]. The Raprl is a fiber bundle that lies posterior to the STN, separated from it by the intervening Zi, and consists of fibers from the mesencephalic reticular formation that project to the thalamus as well as ascending cerebellothalamic fibers. How much of the stimulation of the ZI overflows into the neighboring Raprl is unknown and may vary according to individual electrical conductivity of these structures in individual patients. The exact mechanism of how stimulation of PSA suppresses various tremors still awaits further studies to corroborate.

## Conclusion

PSA could potentially be an alternative target for the tremor, particularly for those tremors difficult to be controlled by traditional VIM DBS, including the proximal postural tremor, distal intention tremor, and some cerebellar outflow tremor. The effect of PSA DBS on axial head tremor and vocal tremor also seems to be promising. The adverse effect profile of the PSA appears transient and mild. However, the conclusion is limited by the small numbers of studies so far. More studies, including randomized double-blinded trials comparing the effect of DBS targeting PSA with that targeting VIM, are needed to help us better understand the efficacy and adverse effect profile of the PSA DBS, which could have profound effect on tremor control, particularly for those difficult to be controlled by traditional VIM DBS.

## Competing interests

The author(s) declare that they have no competing interests.

## Authors’ contributions

TX: Drafting and revising the manuscript; JB: Revising the manuscript; PW: Revising the manuscript. All authors read and approved the final manuscript.
